# Methanolic extract of *Agerantum conyzoides* exhibited toxicity and growth disruption activities against *Anopheles gambiae sensu stricto* and *Anopheles arabiensis* larvae

**DOI:** 10.1186/s12906-016-1464-7

**Published:** 2016-11-22

**Authors:** Jackson Mbithi Muema, Sospeter Ngoci Njeru, Céline Colombier, Rose Muthoni Marubu

**Affiliations:** 1 Department of Biochemistry, Jomo Kenyatta University of Agriculture and Technology, P.O. Box 62000-00200, Nairobi, Kenya; 2 Department of Medicine, Kisii University, P.O. Box 408-40200, Kisii, Kenya; 3 École Supérieure d’Agricultures Institute, 55, Rue Rabelais, B.P 30748 49007 Angers Loire, France; 4 International Centre of Insect Physiology and Ecology, P.O. Box 30772-00100, Nairobi, Kenya

**Keywords:** *Agerantum conyzoides*, Malaria, *Anopheles gambiae s.s*, *Anopheles arabiensis*, Larvicidal activity, Vector control, Growth disruption

## Abstract

**Background:**

Vector control remains the mainstay to effective malaria management. The negative implications following persistent application of synthetic insecticides geared towards regulation of mosquito populations have necessitated prospection for ecofriendly effective chemistries. Plant-derived compounds have the potential to control malaria-transmitting mosquito populations. Previously, *Agerantum conyzoides* extracts have demonstrated toxicity effects on disease-transmitting mosquitoes. However, their efficacy in controlling Afrotropical malaria vectors remains unclear. Herein, the toxicity and growth disruption activities of crude methanolic leaf extract of *A. conyzoides* on *Anopheles gambiae sensu stricto* and *An. arabiensis* larvae were assessed*.*

**Methods:**

Late third (L3) instars of *An. gambiae s.s* and *An. arabiensis* larvae were challenged with increasing doses of crude methanolic extract of *A. conyzoides.* The larval mortality rates were recorded every 24 h and the LC_50_ values determined at their associated 95% confidence levels. ANOVA followed by Post-hoc Student-Newman-Keuls (SNK) test was used to compare results between treatment and control groups. Phytochemical profiling of the extract was performed using standard chemical procedures.

**Results:**

Treatment of larvae with the methanolic extract depicted dose-dependent effects with highest mortality percentages of ≥ 69% observed when exposed with 250 ppm and 500 ppm for 48 h while growth disruption effects were induced by sublethal doses of between 50–100 ppm for both species. Relative to experimental controls, the extract significantly reduced larval survival in both mosquito species (ANOVA, F_(8,126)_ = 43.16776, *P <* 0.001). The LC_50_ values of the extract against *An. gambiae s.s* ranged between 84.71–232.70 ppm (95% CI 81.17–239.20), while against *An. arabiensis* the values ranged between 133.46–406.35 ppm (95% CI 131.51–411.25). The development of the juvenile stages was arrested at pupal-larval intermediates and adult emergence. The presence of alkaloids, aglycone flavonoids, triterpenoids, tannins and coumarins can partly be associated with the observed effects.

**Conclusion:**

The extract displayed considerable larvicidal activity and inhibited emergence of adult mosquitoes relative to experimental controls, a phenomenon probably associated with induced developmental hormone imbalance. Optimization of the bioactive compounds could open pathways into vector control programmes for improved mosquito control and reduced malaria transmission rates.

## Background

The operational scale up of indoor residual spraying (IRS), long-lasting insecticide treated nets (LLINs) and artemisinin-based combined therapy (ACT) over the last decade progressively declined transmission rates of malaria to vulnerable children and pregnant women [1]. An estimate of 69% fewer malaria cases has been reported in sub-Saharan Africa between 2001–2015 following the widespread deployment of the three key interventions [1]. However, evolution of resistance to the active ingredients of these tools and little consideration of developing new complementary compounds targeting the ever changing behavioral traits of Afrotropical malaria vectors have greatly challenged efforts geared to bring malaria under control [2–4]. Nevertheless, vector control forms the integral platform of integrated malaria management aimed to reduce malaria reproduction rate to less than 1 [5, 6].

Larviciding, a less-practiced component of integrated vector management (IVM) and larval source management (LSM), appears a promising approach of suppressing both indoor and outdoor feeding mosquito populations [7–9]. Impressive stories from Brazil and Egypt following its impactful malaria eradication motivates its revival [10] with current operation in Kenya [11], The Gambia [8], Burkina Faso, Benin [12] and Tanzania [13]. Low immobility, confinement to shallow water bodies, susceptibility to chemical attacks and less chances of developing resistance favor this vector control approach [14, 15]. Additionally, manipulation and/or modification of larval habitat bio-physicochemical parameters negatively influence vector competence of resultant mosquitoes suggesting a feasible target of mosquito control [16, 17]. For millennia, mosquito control has considerably relied on chemicals that inevitably reduced environmental quality and facilitated emergence of resistant mosquito strains a phenomenon that has limited their continued reliance, prompting for alternative chemistries [18].

One feasible way of averting the aforementioned drawbacks is prospecting for novel compounds with less environmental impacts and selectively toxic to target arthropods [19, 20]. In addition to being a rich source of bioactive pharmacophores, plants produce allelochemicals with great potential of controlling crop pests and disease-transmitting vectors [21, 22]. Among these are essential oils documented to repel nuisance human biting mosquitoes in addition to inducing toxicity to developing juveniles [23–25]. Non-volatiles, for instance, Azadirachtin and its derivatives from neem tree and plant-based ecdysteroidal analogs potentially inhibit larval development and adult emergence terminating insect metamorphosis immaturely [25, 26]. Additionally, these compounds induce growth disruption effects resulting into mortalities and non-viable females incapable of lineage progression [27–29]. Taken together, plant-derived compounds are promising sources of effective insecticides with meager chances of resistance development afforded by multimodal targets [30, 31].

*Agerantum conyzoides* L. is an *Asteraceae* herbaceous weed that grows in many countries worldwide. Ethnopharmacological surveys of this polyherbal plant have documented biological activities such as analgesic, anti-inflammatory, purgative, febrifuge, anti-asthmatic, antibacterial, antifungal, antispasmodic, anti-diarrhoeic, headache relief, antihelmintic and nematicidal [32, 33]. Phytochemically, the plant contain various bioactive compounds including alkaloids, coumarins, flavonoids, tannins and essential oils [34, 35]. Of considerable interest, the plant extracts have shown detrimental effects on survival, development and adult emergence of mosquitoes such as *Aedes albopictus* [36], *Culex quinquefasciatus* [37], *Aedes aegypti* and *Anopheles stephensi* [38] which has been attributed to possibility of compounds with anti-juvenile hormone activity. However, effectiveness of the plant extracts to control the principal Afrotropical malaria vectors *An. gambiae sensu stricto* and *An. arabiensis* remain obscure. Therefore, we sought to evaluate the larvicidal and developmental disruption effects of *A. conyzoides* against *An. gambiae s.s* and *An. arabiensis*. Our findings demonstrate for the first time to the best of our knowledge that, the methanolic leaf extract of *A. conyzoides* had considerable larvicidal and development inhibition activities in a dose-dependent manner against Afrotropical malaria vectors. In addition, we identified alkaloids, aglycone flavonoids, triterpenoids, tannins and coumarins as phytochemicals that were associated with the observed bioactivities.

## Methods

### Collection of plant material

Ethnobotanical survey was conducted to identify the existing gaps of *A. conyzoides* based on chemotaxonomic criterion. *A. conyzoides* leaf samples were collected from Shinyalu in Kakamega County of western Kenya in September, 2015. The plant was identified by Mr. Thomas Mbasi an ethnobotanical specialist at Kakamega Forest Reserve, and a voucher specimen deposited at the same institution. The leaf samples were packaged in a non-sterile adsorbent paper and transported to the laboratory for processing, extraction and bioactivity assays.

### Preparation of crude leaf extracts

The leaves were shade-dried at room temperature to a constant weight and ground into a fine powder using an electric miller (Retsch Muhle, Haan, Germany). A 100 g of the leaf powder was subjected to methanol (Sigma Aldrich, St. Louis, USA) (2 L) extraction using soxhlet extraction technique for 8–10 h. After cooling to room temperature, the resultant extract was concentrated using a rotary evaporator (Laborota 4000 efficient, Heidolph, Germany) at a temperature of 40 °C under vacuum and stored at −20 °C until required for larvicidal bioassays.

### Phytochemical profiling

The principal bioactive secondary metabolites including alkaloids, terpenoids, steroids, aglycone flavonoids, tannins and coumarins were assayed using standard procedures [39].

### Mosquito colony culture

The experiments were carried out with *An. gambiae s.s* and *An. arabiensis* larvae from a colony maintained at the International Centre of Insect Physiology and Ecology (*icipe*) Insect Mass Rearing Unit. The larvae were separately reared under laboratory conditions of water temperature (28 ± 2 °C), relative humidity of 55–60% and 12:12 h (light: dark) photoperiod. The larvae were reared in large plastic pans (37 × 31 × 6 cm) with distilled water at densities of 200–300 per pan and supplemented with artificial diet Tetramin® fish meal (Tetra GmbH, Melle, Germany). The rearing water was replaced with fresh water and diet after every two days. Pupae were held in plastic cups and transferred into standard 30 × 30 × 30 cm rearing cages. Emergent adults were provided with 10% sucrose solution contained in a glass tube (2 × 8 cm) connected to a paper tube as a wick. Female mosquitoes were blood-fed on restrained Swiss albino mice about 4–5 days post-emergence and provided with oviposition plastic containers (11.5 cm in diameter and ~ 6.2 cm in depth, lined interiorly with a piece of filter paper as oviposition site) for egg collection 2–3 days after blood meal. The eggs were air-dried under insectarium conditions ready for colony cycle maintenance.

### Larvicidal bioassays

The bioassays were conducted in accordance with the World Health Organization guidelines for testing larvicides [40] and adopted by Nyamoita et al., [41]. Crude methanolic extract (250 mg, 125 mg, 50 mg and 25 mg) was separately dissolved in 1 ml of analytical grade ethanol (Fisher Scientific, Loughborough, UK) and diluted with 499 ml of distilled water to make a 500 ml stock solution. This was then dispensed into five beakers each 100 ml to make the required concentrations of 500 ppm, 250 ppm, 100 ppm and 50 ppm, respectively. The bioassays were performed with batches of 20 (*n* = 20) late third instar larvae (L3) of *An. gambiae* and *An. arabiensis* per beaker. The assays were replicated five times and ran simultaneously yielding a total of 100 larvae for each dosage. The control was set up with 1 ml of ethanol diluted in 499 ml distilled water and dispensed into five beakers. The larvae were fed on TetraMin® fish meal (Tetra GmbH, Melle, Germany) during the testing period. Larval mortality (at higher doses) and morphological defects (at lower doses) were monitored at intervals of 24 h until the death of the last larva or emergence of an adult. Larvae were considered dead if they remained irresponsive within a span of two minutes when gently probed with a pipette. The number of the dead larvae was expressed as average percentage mortality for each concentration relative to negative controls.

### Statistical analysis

The corrected larval mortality was expressed as % mean ± S.D of experimental replicates for each dosage of the extract. Dose-responses were analyzed by non-linear regression and half-maximal lethal concentrations (LC_50_) estimated at their associated 95% confidence levels using R software version 3.2.3 [42]. Significant differences between treatment means were established with analysis of variance (ANOVA) followed by Student-Newman-Keuls (SNK) test and *p* values of less than 0.05 considered statistically significant. Graphs were designed using GraphPad Prism version 7.01 for Windows (GraphPad Software, San Diego, California, USA).

## Results

### Phytochemical analysis

Qualitative analysis of *A. conyzoides* leaf extract revealed presence of alkaloids, aglycone flavonoids, triterpenoids, coumarins and tannins (Table 1).

**Table 1:** Phytochemical constituents of crude leaf extract of *A. conyzoides*

Phytochemical constituent	Absence/presence
Alkaloids	+
Flavonoids	+
Tannins	+
Coumarins	+
Terpenoids	+

+ (Presence), − (Absence)

### Effect of the crude extracts on larval survival

**Fig. 1:**
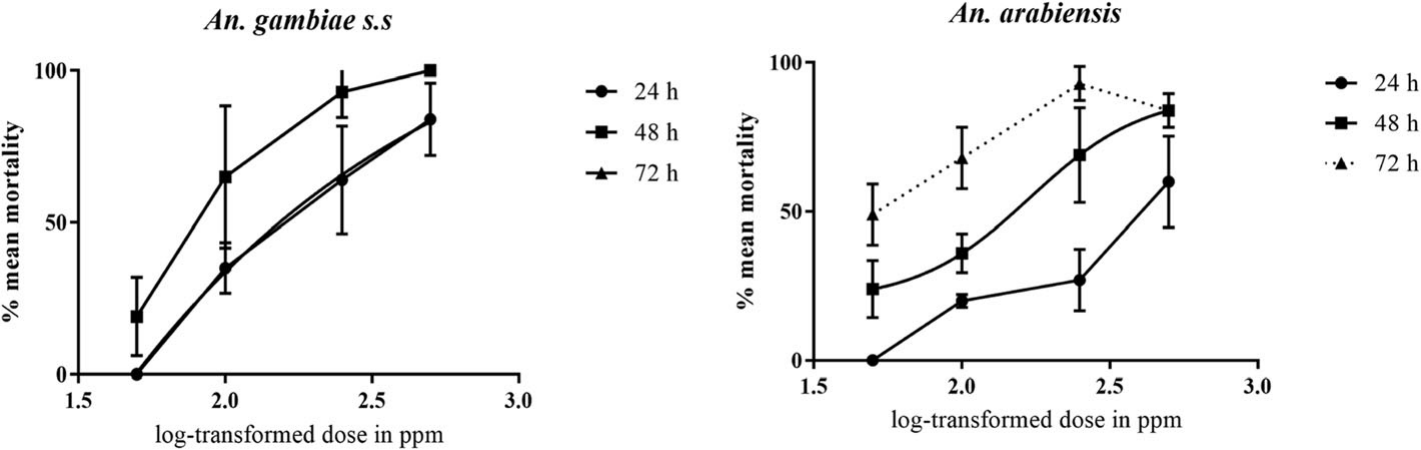
Dose-response curves for *An. gambiae s.s* and *An. arabiensis* larvae to *A. conyzoides* extract for 24 h, 48 h and 72 h post exposure. Doses are log-transformed and each point on the plots represents percentage mean (± S.D) larval mortality of 5 replicates for each dose of the extract

The toxicity of crude methanolic extract of *A. conyzoides* against late 3^rd^ instars of *An. gambiae s.s* and *An. arabiensis* was evaluated. The toxicity of the extract was demonstrated to be dose-dependent with high doses of 250 ppm and 500 ppm showing ≥ 69% larval mortality at 48 h post-exposure compared to the lower doses of 50 ppm and 100 ppm which gave < 50% larval mortality (Table 2). Maximum larval mortality (100%) was recorded at 500 ppm on exposure to *An. gambiae s.s* larvae for 48 h with only 88% attained against *An. arabiensis*. A 100% larval survival was noted in the negative control group for the entire analysis period. Relative to controls, the extract significantly reduced survival rates of *An. gambiae s.s* (ANOVA, F_(4,70)_ = 115.5534, *P <* 0.001) and *An. arabiensis* larvae (ANOVA, F_(4,70)_ = 31.7382, *P <* 0.001). There was significant susceptibility difference between the two mosquito species to the extract (ANOVA, F_(8,111)_ = 25.6398, *P <* 0.001). A time- and dose-dependent reduction in survival rates of extract-challenged mosquito larvae was demonstrated in Fig. 1.

**Table 2:** Mean larval mortality evoked by the *A. conyzoides* extract at different concentrations against 3^rd^ instar larvae of *An. gambiae s.s* and *An. arabiensis*

Time	% mean mortality ± S.D^a^					Lethal concentration (ppm)	
	500 ppm	250 ppm	100 ppm	50 ppm	Control	LC_50_	95% CI
*An. gambiae s.s* larvae							
24 h	84 ± 11.94	64 ± 17.82	35 ± 8.37	0 ± 0.00	0 ± 0.00	232.70	228.85–239.20
48 h	100 ± 0.00	93 ± 8.37	65 ± 23.45	19 ± 12.94	0 ± 0.00	84.31	81.17–90.88
72 h	100 ± 0.00	93 ± 8.37	65 ± 23.45	19 ± 12.94	0 ± 0.00	84.31	81.17–90.88
*An. arabiensis* larvae							
24 h	60 ± 15.41	27 ± 10.37	20 ± 2.24	0 ± 0.00	0 ± 0.00	406.35	403.56–411.25
48 h	88 ± 5.70	69 ± 15.97	36 ± 6.52	24 ± 9.62	0 ± 0.00	133.46	131.51–136.16
72 h	88 ± 5.70	93 ± 5.70	68 ± 10.37	49 ± 10.25	0 ± 0.00	133.46	131.51–136.16

Data expressed as % mean mortality ± standard deviation (S.D), LC_50_- lethal concentration that killed 50% of mosquito larvae population, *CI* Confidence Interval

^a^Mortality means are significantly different at *p ≤* 0.05 (Student-Newman-Keuls test)

### Developmental disruption effects

**Fig. 2:**
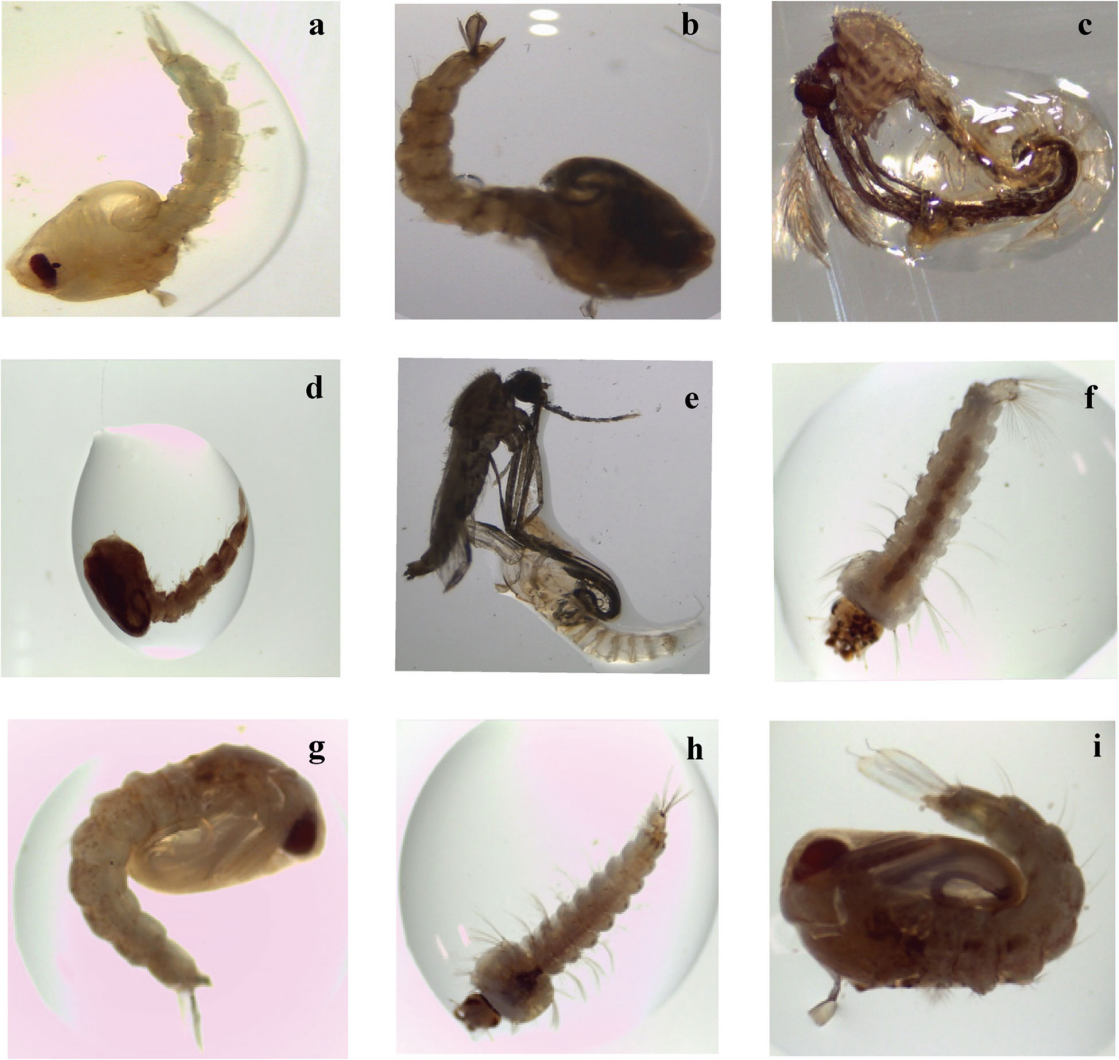
Development disruption effects of *A. conyzoides* extract to *An. gambiae s.s* and *An. arabiensis*. **a** Demelanized *An. gambiae s.s* larvae (**b**) Abnormal *An. gambiae s.s* larval-pupal intermediate (**c**) Arrested adult emergence in *An. gambiae* (**d**) Abnormal *An. arabiensis* larval-pupal intermediate (**e**) Failed adult emergence in *An. arabiensis* (**f**) *An. gambiae s.s* control larvae (**g**) Normal *An. gambiae s.s* larval-pupal intermediate (**h**) *An. arabiensis* control larvae (**i**) Normal *An. arabiensis* larval-pupal intermediate (Light microscopy visualization conducted at magnification 25×)

Besides lethality of high doses, the extract remarkably accelerated the growth of larvae into pupae resulting into incomplete melanization and abnormal dead larval-pupal intermediates as depicted in Fig. 2. At sublethal doses of 50 ppm and 100 ppm, molting continued normally but the development of the immature stages was greatly affected. Microscopic examination of the dead immature stages at 25× magnification revealed morphological defects evident as:- abnormal dead larval-pupal intermediates and emergent adults with mouthparts and wings folded within the pupal exuvium (Fig. 2). The adults that luckily emerged from the extract-treated water were unable to escape from the pupal caste and died on the surface of test solution. Overall, the extract at sublethal doses induced prolonged larval phase duration by 7 more days prior to pupation relative to negative controls (2 days).

## Discussion

In search for better insecticides to replace or complement the synthetic insecticides and alleviate resistance pressure on malaria vectors, scientists have turned interests into nature for alternative controls. Many plants have been reported around the globe to have bioactivity against mosquitoes and their multiple targets of actions against mosquitoes assure effectiveness as alternative bio-insecticides. The toxic efficacy of these botanicals against various mosquito vectors vary depending on different factors such as the part of the plant used, method of extraction adopted, solvent used, geographical locality the plant was obtained, the concentration of the extract used and photosensitivity of some plant compounds [43].

In the current study, we challenged late third (L3) instar larvae of *An. gambiae s.s* and *An. arabiensis* with crude extract of *A. conyzoides* to evaluate their responses. Our data demonstrate that the extract had detrimental effects on both survival and development of *An. gambiae s.s* and *An. arabiensis* in a dose-dependent response manner. High dosages of 250 ppm and 500 ppm evoked acute toxicity to the developing larvae while the sublethal doses of 50 and 100 ppm induced developmental disruptions as shown by Fig. 2(b-e). The toxic effect of the plant extract could be attributed to its bioactive phytochemical constituents (Table 1). The LC_50_ values of the plant extract have shown significant potential of controlling *An. gambiae s.s* and *An. arabiensis*. It has been previously reported that the plant extract had larvicidal activity against *Ae. albopictus* [36], *C. quinquefasciatus* [37], *Ae. aegypti* and *An. stephensi* [38] which is similar to our data though slight variation was noted. This could be attributed to the solvents used for extraction, susceptibility differences of mosquito vectors used and geographical differences of the plant. Methanolic extract of *A. conyzoides* leaves was used in the present study and found effective against *An. gambiae s.s* and *An. arabiensis* larvae.

Moreover, phytochemicals extracted from many plant species have been reported to show growth inhibiting effects on the various developmental stages of different mosquito species [27–29, 40, 43]. Various pre-emergent effects such as prolongation of larval instar and pupae durations, inhibition of larval and pupal molting, morphological abnormalities and mortality may occur especially during molting and melanization processes. Developmental disruption effects induced by the plant extracts can be associated with disturbed hormonal balance or interference in chitin synthesis during the molting process. Our data recorded morphological effects on *An. gambiae s.s* and *An. arabiensis* where the immature stages failed to transform into a normal adult leading to eventual death (Fig. 2c and e). Similar results on morphological abnormalities were reported on *An. stephensi, Ae. aegypti* and *C.* quin*quefasciatus* exposed to *A. conyzoides* extract. The phenomenon has been reported by Okunade, [35] as a result of perturbation of hormonal homeostasis by precocene-3,4-epoxide, a metabolite generated by cytochrome P450s in the insect body [44]. The metabolite may either antagonize or agonize the biosynthesis and subsequent release of juvenile hormone, the regulator of insect metamorphosis.

Studies carried out by Nyamoita et al., [41], Nathan et al., [45] and Nathan et al., [46] reported that in addition to their lethality, the secondary metabolites of the botanicals used resulted in protracted larval phase, disrupted growth and malformation of the exoskeleton. Although there was no elongation of gut as observed in [29] and [47], incomplete melanization process was observed in larvae and some pupae examined under light microscopy (Fig. 2). Our data corroborated with that obtained by Ndung’u et al., [28] where limonoids from methanolic extracts of the root of *Turraea mombassana* Hiern (*Meliaceae*) resulted in larval and pupal morphological deformities in *An. gambiae s.s* due to incomplete melanization. Similarly, exposure of *Anopheles stephensi* to extracts of *Melia azedarch* resulted in similar observations [45]. Also, compounds from *Azadirachta indica* and *Melia volkensii* (*Meliceae*) extracts induced growth disruption effects to mosquito larvae besides feeding deterrence and toxicity [48]. Studies performed by Govindachari et al., [49], Martinez and Van Emden, [50] and Nathan et al., [51] confirmed the above effects of Azadirachtin on insects. Elsewhere, dichloromethane extract of *Hyptis brevis* (*Lamiaceae*) displayed strong growth inhibition on *Spodoptera littoralis* larvae by arresting metamorphosis [52]. The same phenomenon has been reported by Cespedes et al., [53].

Several plant species produce a myriad of bioactive chemicals as part of defenses against herbivory attacks majorly classified as volatile compounds (essential oils) and non-volatiles. The non-volatiles include the alkaloids, flavonoids, terpenoids, glucosinolates, cyanogenic glycosides, phenolic acids among others [31]. Majority of these non-volatiles particularly phytoecdysteriods, phytojuvenoids and anti-juvenile hormones act as insect growth regulators (IGRs) reducing survival rates and development of insects upon ingestion [54]. Previous reports indicate various insecticidal compounds isolated and identified from *A. conyzoides* extracts such as steroids, flavonoids, coumarins, pyrrolizidine alkaloids, triterpenoids, and chromenes [36, 55–57]. In this regard, phytochemical analysis revealed presence of main compounds such as alkaloids, terpenoids (e.g. precocene I and precocene II) [56], flavones (e.g. ageconyflavones A, B and C) [57], coumarins and tannins which equally agree with these reports. All these compounds may act in a concerted manner to nonspecifically induce toxicity to insects. More specifically, precocenes (terpenoids) have been reported to be anti-juvenile hormone, accelerating the development of insects and inducing dwarfness associated with low survival rates [43]. Phytochemicals that agonize or antagonize the effects of insect development hormones have been reported to be good bio-pesticides [53]. These compounds disrupt the normal metabolism of the insect hormones during the development of the juveniles leading to failure of adult emergence [55].

Two important insect developmental hormones that interplay are 20-hydroxyecdysone (20-E) and juvenile hormone (JH) [58]. It is the balance in levels of these two hormones that define the outcome of each developmental transition [59]. Ligand-binding to the insect juvenile receptor complex disrupt insect endocrine signaling and regulation causing abnormal development and lethality [21]. The accumulation of these plant compounds above threshold levels disrupt the insects’ developmental progression culminating into premature death or failure to emerge as a normal adult [60]. The active compounds from *A. conyzoides* extract induced toxicity and growth inhibition effects to developing mosquito larvae and could potentially be isolated for formulating effective mosquito control agents. Further, identification of molecular targets, ligand docking and simulation assays accompanied by field applications could be pursued for improved mosquito control.

## Conclusions

The findings of our study showed promising larvicidal and development disrupting effects of *A. conyzoides* extract on the main Afrotropical malaria vectors, *An. gambiae s.s* and *An. arabiensis.* Phytochemicals present within the extract including alkaloids, aglycone flavonoids, triterpenoids, tannins and coumarins were associated with the observed experimental effects. They have potential of being used as insecticides for controlling mosquito populations around human dwellings by targeting the immature stages. Noteworthy, prior to commercial application of this botanical larvicide, factors such as safety of non-targets and beneficial organisms, efficacy in actual field conditions, and residual half-life must be put into consideration.

## Abbreviations

ACT: Artemisinin-based combined therapy
*icipe*: International Centre of Insect Physiology and Ecology
IRS: Indoor residual spraying
IVM: Integrated vector management
LLINs: Long-lasting insecticide treated nets
LSM: Larval source management
